# Forensic life-threat assessments using trauma scoring in single stabs to the trunk

**DOI:** 10.1007/s00414-026-03781-6

**Published:** 2026-04-10

**Authors:** Maria Berg von Linde, Stefan Acosta, Ardavan M. Khoshnood, Carl Johan Wingren

**Affiliations:** 1 Unit for Forensic Medicine, Department of Clinical Sciences Malmö, Faculty of Medicine, Lund University, Sölvegatan 25, Lund, 223 62 Sweden; 2 Unit for Forensic Medicine, Swedish National Board of Forensic Medicine, Sölvegatan 25, Lund, 223 62 Sweden; 3 Vascular Centre, Department of Cardiothoracic and Vascular Surgery, Skåne University Hospital, Malmö, Sweden; 4 Department of Clinical Sciences Malmö, Lund University, Ruth Lundskogsgatan 10, Malmö, 205 02 Sweden; 5 Department of Emergency Medicine, Skåne University Hospital Malmö, Malmö, Sweden; 6 Department of Clinical Sciences Malmö, Clinical Research Centre, Lund University, CRC 91-12, Box 50332, Malmö, 202 13 Sweden; 7 Department of Forensic Medicine, University of Copenhagen, Frederik V’s Vej 11, Copenhagen, 2100 Denmark

**Keywords:** sharp force, life-threat, trauma score, forensic medicine

## Abstract

**Introduction:**

Forensic assessments of whether an injury would have been life-threatening often rely on empirical knowledge rather than evidence-based criteria. To address this gap, we applied the validated trauma scoring system New Injury Severity Score (NISS) to estimate mortality risk along the natural course of single stab injuries to the trunk.

**Methods and materials:**

We included 408 survivors and 139 fatalities with single stab injuries to the trunk, using data from registries maintained by the Swedish National Board of Forensic Medicine. Organ and vessel damage in survivors, stratified by intervention complexity; and fatalities, stratified by time until death, were presented. The predictive performance of NISS was estimated using logistic regression and Receiver Operating Characteristic (ROC) analysis.

**Results:**

NISS was strongly associated with intervention complexity among survivors and demonstrated excellent performance in predicting fatal outcomes without lifesaving treatment (area under the curve (AUC) = 0.94, 95% CI 0.92–0.96), with optimal discrimination at NISS ≥ 20 (Youden’s Index = 0.72).

**Discussion and conclusion:**

To increase the evidence base in forensic life-threat assessment, we suggest applying NISS as a complement to the expert opinion, as it offers potential for establishing a transparent and systematic tool that increases the rule of law. However, the tool needs to be validated in other settings and for multiple injuries.

**Supplementary Information:**

The online version contains supplementary material available at 10.1007/s00414-026-03781-6.

## Introduction

In investigations of violent crimes with bodily injuries, clinical forensic examinations can be requested by police to document injuries on victims and perpetrators, and to assess the causation and severity of said injuries. Injury severity assesses whether the injuries would have been life-threatening without medical intervention. The assessment is important for establishing the legal severity of the crime [1, 2], and is included in judicial decisions when sentencing convicted offenders for assault [3, 4]. These forensic assessments are generally informed by expert opinions, often provided by medical professionals such as forensic pathologists, and are often grounded in clinical experience rather than in evidence-based data on population outcomes for the observed injuries.

A consensus document on life-threatening injury types prepared by the Swedish National Board of Forensic Medicine considers injuries that perforated the pleura and/or damaged thoracic or abdominal organs to be life-threatening [5]. Also, according to international consensus in forensic medicine, injuries which require emergency treatment, including surgery or blood transfusion [1], or lead to impairment of the integrity of internal organs, body cavities, major blood vessels and/or nerves, are considered life-threatening [6]. Despite these general guidelines, there is a need for a standardised, evidence-based method to estimate forensic life-threatening injuries to better support a fair penal system; ideally one providing not only categorical assessments but also a numeric scale [3, 7].

In trauma management and epidemiological research, trauma scoring systems are applied for predicting mortality risk. The anatomical scoring systems Injury Severity Score (ISS) and its modification, the New Injury Severity Score (NISS), are widely used to quantify trauma severity [8]. The ISS is calculated by summing the squares of the highest Abbreviated Injury Scale (AIS) score in each of the three most severely injured body regions, whereas the NISS instead sums the squares of the three most severe injuries regardless of body region, thereby accounting for multiple serious injuries within the same anatomical area [8]. The NISS was introduced as a modification of the ISS to address limitations of the original system, particularly its inability to account for multiple severe injuries occurring within the same body region [8]. By allowing the three most severe injuries to be included irrespective of anatomical region, the NISS has been shown to improve mortality prediction compared with ISS [8]. In clinical trauma research, NISS is widely used for outcome prediction in both blunt and penetrating trauma [8]. This score may be particularly relevant for penetrating injuries such as stab wounds, where several critical structures within the same anatomical region may be affected by a single injury mechanism.

However, these scoring systems are based and validated on hospitalised populations receiving medical interventions when required [9, 10], rather than the natural course of the injuries, as is the focus in forensic life-threat assessments. Consequently, these populations include survivors who underwent medical interventions, lowering the risk of mortality. An alternative approach for the evidence-based assessment of life-threat in the forensic setting has been proposed by one of the authors (Wingren CJ), using spleen injury cases for which survivors of life-saving interventions were combined with fatal cases to estimate the mortality risk in the natural course of an injury [3].

We evaluated the predictive performance of NISS for fatal outcome in the natural course and to estimate the mortality risk of specific organ and vascular damage, in a forensic population with a single stab to the trunk using both surviving victims and an autopsy population.

## Materials and methods

### Study populations

Autopsy cases and survivors of single stab injuries to the trunk aged 15 years and older were identified in the autopsy registry between the years 2010 and 2021, and in the registry of clinical forensic medicine examinations between 2016 and 2021, respectively. These registries are maintained by the Swedish National Board of Forensic Medicine.

The methodology for selecting the autopsy population is explained in detail in a previous study based on the same population [11]. During the twelve-year study period, a total of 65,265 forensic autopsies were recorded in the national forensic autopsy registry. To summarise, 474 autopsy cases were initially identified using the International Classification of Diseases 9th edition (ICD-9) codes for sharp force injuries and causes of death involving injuries to the trunk. We included homicidal and suicidal deaths where a single stab injury to the trunk was the primary cause of death. Cases with accidental or undetermined manner of death were excluded. Also, cases in which the extent of the stab injuries could not be reliably determined because of severe fire damage or decomposition were excluded. After manual review, 139 cases (94 homicides and 45 suicides) met the inclusion criteria.

The population of assault survivors was identified in a registry of clinical forensic examinations consisting of police-reported cases in which forensic assessments of the related injuries were requested by police. During the six-year study period, the registry contained 27,640 clinical forensic examination reports. In Sweden, clinical forensic reports are issued by forensic pathologists, and typically include findings from the forensic body examination, and are supplemented, when available, by medical records and photographic documentation of injuries. In many cases a forensic examination is not performed, and in such cases the forensic report is based on medical records and photographs, if available.

To identify relevant cases from the registry, we searched the clinical forensic reports using Swedish terms representing the following keywords: “stab wound”, “knife stab”, “sharp edged”, “pleura”, “stab channel”, “stabbing”, “knife-like”, “knife slash”, “knife cut”. In total, 9162 reports were identified. The first author of the paper manually reviewed these reports and included 408 survivors with a single stab injury to the trunk.

Among the survivors and the autopsy cases, sharp injuries were characterised by stab wounds, or a combination of stab and incised wounds. Stab wounds were defined as sharp force injuries in which the depth of the wound channel exceeds its length on the skin surface, whereas incised wounds are characterised by a surface length greater than the wound depth. A single stab wound in the skin associated with more than one wound channel, was still considered a single stab and was included in the study. Anatomically, we included injuries if the entrance wound was located on the trunk. The anterior trunk was defined as the area between the clavicles superiorly and the pubic bone inferiorly. The axillary regions were defined from the axilla superiorly to the level of the crista iliaca inferiorly. The posterior trunk was defined from the first thoracic vertebra superiorly to the last lumbar vertebra inferiorly. Cases involving more than one stab injury and cases with additional moderate to severe blunt force injuries were excluded, while cases with additional superficial incisive wounds, with a depth that did not exceed subcutaneous tissue, and/or minor blunt force injuries, such as bruises or superficial lesions, were included.

### Collection of variables

Data regarding internal injuries, injury severity and medical interventions were extracted from autopsy reports and clinical forensic reports, respectively, and from paramedic and medical records.

The ages (years) of the survivors and the autopsy cases were defined either at the time of the assault or at the time of death. Age was included as a continuous variable and dichotomised into categories of (i) ≥ 60 years and < 60 years.

Sex was defined from their designation in the police reports.

Major bleeding was defined as a documented loss of ≥ 31% of estimated blood volume, corresponding to Class III–IV haemorrhage (moderate to severe hypovolemic shock) according to the American College of Surgeons’ *Advanced Trauma Life Support* classification [12]. In cases where body weight was not documented, major bleeding was alternatively defined as an estimated blood loss of ≥ 1500mL and/or the administration of blood transfusion.

Major haemothorax was defined as > 1000mL blood loss in the thoracic cavity, in accordance with the Abbreviated Injury Scale’s (AIS) 2005 guidelines [13].

Tension pneumothorax was defined if explicitly diagnosed as such by the treating physician.

Medical interventions among the injured survivors were classified into: (i) no or minor interventions, (ii) moderate interventions, and (iii) life-saving interventions. No or minor interventions included cases where no medical treatment was administered, sutured injuries, administration of prophylactic antibiotics, and/or exploratory surgery without therapeutic intervention. Moderate interventions included interventional surgery, endovascular therapy and/or blood transfusion, but without clinical signs of major bleeding, major haemothorax, or tension pneumothorax. Life-saving interventions were defined as cases with major bleeding, major haemothorax, and/or tension pneumothorax, which necessitated surgical interventions and/or endovascular therapy.

Autopsy cases were partitioned into: (i) cases found within 24 h after the stab injury occurred; and (ii) cases found more than 24 h after injury. The information about time was obtained in the police reports based on information on when the victim was last seen alive, and presumed unharmed, and when the victim was found dead.

We defined: (i) fatal injuries as either injuries in survivors that required life-saving interventions, or autopsy cases where the victim was found dead within 24 h of the stab injury; and (ii) non-fatal injuries like those in survivors requiring no, minimal, or moderate medical interventions. Autopsy cases where the victim was found dead more than 24 h after injury were excluded from the classification of fatal and non-fatal injuries, as such outcomes may reflect delayed complications (such as infections), rather than the immediate lethality of the injury itself. This group was excluded to ensure comparability between injuries that were immediately life-threatening with an expected fatal outcome absent medical intervention, and those who were initially stable enough to not require life-saving interventions upon arrival at the emergency department.

Injuries to organs and vessels were categorised into: (i) no organ or vessel injuries, (ii) injuries to only one organ or vessel, and (iii) injuries to more than one organ and/or vessel. If a stab injury involved the parenchyma of an organ and a named vessel in the same organ, the injury was categorised as (iii) injuries to more than one organ and/or vessel. Injuries to smaller and superficial structures, such as the skin, subcutis, muscles, pleura, pericardium, peritoneum, diaphragm, omentum, mesentery, or vascular branches were defined as injuries involving no organs or vessels.

The cases with no organ or vessel injuries were also divided into: (i) superficial, not penetrating into the thoracic or abdominal cavity; penetrating (ii) the thoracic cavity; (iii) the abdominal cavity; or (iv) thoracic and abdominal cavity.

Injuries to only one organ or vessel were partitioned into injuries to: (i) intestines, (ii) stomach, (iii) kidney, (iv) spleen, (v) liver, (vi) lung, (vii) vessel, and (viii) heart.

Injuries to the intestines and stomach were categorised as: (i) superficial without perforation and (ii) with perforation into the intestinal lumen or the gastric cavity, respectively.

Injuries to a kidney were divided into (i) ≤1 cm the parenchymal depth, and (ii) >1 cm parenchymal depth, and injuries to the spleen and liver were divided into (i) ≤ 3 cm parenchymal depth and (ii) >3 cm parenchymal depth, in accordance with the AIS 2005 guidelines [13].

Lung injuries were divided into: (i) superficial non-penetrating injuries to one lobe (ii); perforating injuries to one lobe, defined as injuries passing completely through the lung parenchyma; (iii) injuries to two lobes, either both superficial or with only one lobe perforated; and (iv) injuries perforating two lobes.

Injuries to all anatomically named vessels were recorded from autopsy reports and medical records.

Cardiac injuries were categorised into: (i) superficial, non-perforating injuries; (ii) injuries perforating right or left atria; and (iii) injuries perforating right or left ventricles in accordance with AIS 2005 guidelines [13].

We scored the severity of the stab injuries using the AIS. Only injuries that were clearly documented in autopsy reports, clinical forensic reports, medical records, or imaging findings were included. In cases where the extent of an injury or bleeding could not be reliably determined, the injury was coded as minor and the bleeding as “not further specified”. The same principle was applied to all organ and vessel injuries described above. Detailed descriptions of the defined criteria for all the AIS codes that we used are presented in a previous study based on an autopsy population with single stabs to the trunk [14]. The three highest scores of AIS, within each case, were documented to calculate the NISS ($\:{\mathrm{A}\mathrm{I}\mathrm{S}}^{2}+{\mathrm{A}\mathrm{I}\mathrm{S}}^{2}+{\mathrm{A}\mathrm{I}\mathrm{S}}^{2}=\mathrm{N}\mathrm{I}\mathrm{S}\mathrm{S}$) [8]. The NISS score ranges from 1 to 75, predicting an increased risk of mortality with a higher score value [8]. The score was categorised into intervals of: (i) minor (NISS ≤ 8); (ii) moderate (NISS 9−15); (iii) severe (NISS 16−24); and (iv) critical injuries (NISS 25−75). To facilitate practical application of the NISS methodology, illustrative examples of AIS coding and NISS calculation are provided in Supplementary Table S1.

### Statistics

Categorical variables were presented in numbers and percentages, and the differences between survivors—stratified by medical interventions (no/minimal interventions, moderate interventions and life-saving interventions)—and fatalities—stratified by the body having been found either within 24 h–24 h after the injury occurred—were analysed using the Fisher’s exact test and chi-square test. Age was presented using median and range, and comparisons between survivors and fatalities were tested using the Mann–Whitney U test. *P* < 0.05 was considered statistically significant.

The distribution of NISS among survivors, stratified by increasing complexity of medical interventions, and fatalities, stratified by whether the body was found before or after 24 h from the injury was inflicted, were presented in median and range. Differences between the distribution of NISS was tested using Kruskal–Wallis test.

The association between the predictors of age and sex and the outcome ‘fatal injuries’ was analysed using a binary logistic regression model, adjusted for the categories of NISS (minor, moderate, severe and critical injuries). The dependent variable was fatal injuries using non-fatal injuries as reference. Odds ratios (OR) were expressed with 95% confidence intervals (CI).

The diagnostic performance of NISS in predicting fatal injuries was assessed using Receiver Operating Characteristic (ROC) analysis. Non-fatal injuries served as reference group. Area under the curve (AUC) values with 95% CI were calculated to evaluate discriminatory performance. The AUC values were interpreted as follows: 0.90–1.0 = excellent; 0.80–0.90 = good; 0.70–0.80 = fair; 0.60–0.70 = poor; 0.50–0.60 = failure [15].

Sensitivity, specificity, and Youden´s Index (Sensitivity + Specificity − 1), each with 95% CI, were calculated for all NISS cut-offs after cross-tabulation of fatal injuries versus non-fatal injuries [16].

Data analyses were performed using IBM SPSS Statistics Premium 28 (SPSS, Chicago, IL, USA).

## Results

### Population characteristics

A total of 547 cases were included; 408 survivors and 139 fatalities (Table 1). Males dominated among both groups, accounting for 92.4% of survivors and 89.2% of fatalities. Cases with fatal injuries were significant older than survivors, with median ages of 40 years and 31 years, respectively.

**Table 1:** Population

	Survivors, *n* (%)	Fatalities, *n* (%)	*P*-value
Number of cases	408 (100)	139 (100)	
Males	377 (92.4)	124 (89.2)	
Females	31 (7.6)	15 (10.8)	0.3
Age in years, median (range)	31 (15–79)*	40 (15–90)	< 0.001

Sex was presented in numbers and percentages based on the column categories of survivors and fatalities and group difference between survivors and fatalities was estimated using Fisher´s exact test. Age was presented as median and range in years, and group difference between survivors and fatalities was estimated using the Mann–Whitney U test

*Missing values: age n =1 among survivors

### Organ and vessel damages

The distribution of organ and vessel injuries in survivors, stratified by level of medical intervention; and fatal cases, stratified by the body having been found within or after 24 h, is summarised in Table 2. There were a few cases in both of the autopsy groups (*n* ≤ 3, respectively) and the survivor group requiring life-saving interventions (*n* = 10, 4.3%) without any documented injuries to major internal organs or named vessels, involving cases of laceration to the pectoral muscle, pneumothorax, haemothorax, hemopneumothorax, laceration of the diaphragm, omentum, mesentery, and those limited to perforations of the abdominal wall. Among all injuries that perforated the abdominal wall with no internal injuries to major organs or named vessels (*n* = 26), a total of four cases led to major bleeding with need for life-saving interventions or fatal outcomes (*n* = 4/26, 15.4%).

**Table 2:** Organ and vessel damage among survivors categorised by complexity of medical interventions and fatalities categorised by time of stabbing until death

	All cases	Survivors			Fatalities		*P*-value
		No/minimal interventions *n* = 179	Moderate interventions *n* = 170	Life-saving interventions *n* = 59	Found within 24 h,*n* = 115	Found after 24 h,*n* = 24	
No organ or vessel injuries	230 (100)	156 (67.8)	61 (26.5)	10 (4.3)	≤ 3 (≤ 1.3)	≤ 3 (≤ 1.3)	
Superficial	127 (100)	121 (95.3)	4 (3.1)	≤ 3 (≤ 2.4)	0 (0)	0 (0)	
Thoracic cavity	67 (100)	17 (25.4)	44 (65.7)	5 (7.5)	0 (0)	≤ 3 (≤ 4.5)	
Abdominal cavity	26 (100)	14 (53.8)	8 (30.8)	≤ 3 (≤ 11.5)	≤ 3 (≤ 11.5)	≤ 3 (≤ 11.5)	
Thoracic and abdominal cavities	10 (100)	4 (40.0)	5 (50.0)	≤ 3 (≤ 30.0)	0 (0)	0 (0)	< 0.001
Intestinal injuries only							
No perforation	11 (100)	≤ 3 (≤ 27.3)	8 (72.7)	0 (0)	0 (0)	0 (0)	
Perforation	25 (100)	0 (0)	19 (76.0)	5 (20.0)	0 (0)	≤ 3 (≤ 12.0)	0.02
Stomach injuries only							
No perforation	≤ 3 (100)	0 (0)	≤ 3 (100)	0 (0)	0 (0)	0 (0)	
Perforation	10 (100)	0 (0)	6 (60.0)	≤ 3 (≤ 30.0)	0 (0)	≤ 3 (≤ 30.0)	0.5
Kidney injuries only							
≤ 1 cm depth	5 (100)	≤ 3 (≤ 60.0)	≤ 3 (≤ 60.0)	0 (0)	0 (0)	0 (0)	
> 1 cm depth	≤ 3 (100)	0 (0)	0 (0)	≤ 3 (100)	0 (0)	0 (0)	0.02
Spleen injuries only							
≤ 3 cm depth	5 (100)	≤ 3 (≤ 60.0)	4 (80.0)	0 (0)	0 (0)	0 (0)	
> 3 cm depth	≤ 3 (100)	0 (0)	≤ 3 (100)	0 (0)	0 (0)	0 (0)	1.0
Liver injuries only							
≤ 3 cm depth	22 (100)	7 (31.8)	12 (54.5)	≤ 3 (≤ 13.6)	≤ 3 (≤ 13.6)	0 (0)	
> 3 cm depth	4 (100)	0 (0)	≤ 3 (≤ 75.0)	0 (0)	≤ 3 (≤ 75.0	0 (0)	0.3
Lung injuries only							
One lobe, no perforation	40 (100)	8 (20.0)	23 (57.5)	5 (12.5)	≤ 3 (≤ 7.5)	≤ 3 (≤ 7.5)	
One lobe, perforation	8 (100)	0 (0)	≤ 3 (≤ 37.5)	0 (0)	4 (50.0)	≤ 3 (≤ 37.5)	
Two lobes, no perforation	≤ 3 (100)	0 (0)	0 (0)	0 (0)	≤ 3 (100)	0 (0)	
Two lobes, perforation	≤ 3 (100)	0 (0)	0 (0)	0 (0)	≤ 3 (100)	0 (0)	0.004
Vessel injuries only							
Aorta	7 (100)	0 (0)	0 (0)	≤ 3 (≤ 42.9)	5 (71.4)	≤ 3 (≤ 42.9)	
Vena cava	≤ 3 (100)	0 (0)	0 (0)	0 (0)	≤ 3 (100)	0 (0)	
Iliac artery	≤ 3 (100)	0 (0)	0 (0)	0 (0)	≤ 3 (100)	0 (0)	
Intercostal artery	≤ 3 (100)	0 (0)	≤ 3 (100)	0 (0)	0 (0)	0 (0)	
Subclavian artery	≤ 3 (100)	0 (0)	≤ 3 (100)	0 (0)	0 (0)	0 (0)	
Axillary vein	≤ 3 (100)	0 (0)	0 (0)	≤ 3 (100)	0 (0)	0 (0)	
Internal thoracic artery	≤ 3 (100)	0 (0)	≤ 3 (100)	0 (0)	0 (0)	0 (0)	0.2
Heart injuries only							
Superficial	≤ 3 (100)	0 (0)	0 (0)	≤ 3 (≤ 100)	0 (0)	≤ 3 (≤ 100)	
Perforation of atrium	≤ 3 (100)	0 (0)	0 (0)	≤ 3 (≤ 100)	≤ 3 (≤ 100)	0 (0)	
Perforation of ventricle	31 (100)	0 (0)	0 (0)	0 (0)	22 (71.0)	9 (29.0)	0.001
Injuries to more than one organ and/or vessel	128 (100)	≤ 3 (≤ 2.3)	21 (16.4)	27 (21.1)	72 (56.3)	6 (4.7)	< 0.001

The subgroups of survivors and fatalities were presented in numbers and percentages based on the row categories of the different organ and vessel injuries. Differences between the groups were estimated using a chi-square test. Cell counts ≤ 3 are not reported in detail for confidentiality

The few organ injuries among survivors requiring no/minimal interventions consisted of superficial injuries to the lungs (*n* = 8), liver (*n* = 7), intestines (*n* ≤ 3), kidneys (*n* ≤ 3), and spleen (*n* ≤ 3).

Injuries perforating the intestines or stomach, without any other internal injuries, were most commonly seen in survivors requiring moderate medical interventions (76.0% and 60.0%, respectively) with a few cases among survivors receiving life-saving interventions (20.0% and *n* ≤ 3, respectively), and fatal cases in which the body was found after more than 24 h (*n* ≤ 3 and *n* ≤ 3, respectively). Also, the majority of cases with injuries isolated to either the spleen, liver, or the lungs were seen in survivors receiving moderate medical interventions (> 80%, > 54.5%, and > 57.5%, respectively).

Vessel injuries without organ injuries occurred in a few cases of survivors requiring moderate intervention, involving injuries to the subclavian artery, internal thoracic artery, and intercostal arteries. Among the survivors requiring life-saving interventions, less than three cases of vessel injuries without organ injuries were seen, involving injuries to the axillary vein and the aorta. A few fatal vessel injuries, without organ injuries, were seen involving the aorta, vena cava, and iliac artery.

Most cases with injuries limited to the heart consisted of ventricular perforations, all of which were fatal.

### New injury severity score

Among survivors, the median NISS were: 1 (range 1–22) among cases which required no or minimal medical intervention; 13 (range 1–29) among cases which required moderate intervention; and 20 (range 4–50) among cases which received life-saving interventions (Table 3). Fatal cases had a median NISS of 75 (range 8–75) among cases found within 24 h, and a median NISS of 50 (range 1–75) among cases that were found 24 h after the injury.

**Table 3:** New Injury Severity Score among survivors categorised by medical interventions and fatalities categorised by time until death

	Survivors			Fatalities		*P*-value
	No/minimal interventions *n* = 179	Moderate interventions *n* = 170	Life-saving interventions *n* = 59	Found within 24 h *n* = 115	Found after 24 h *n* = 24	
NISS, median (range)	1 (1−22)	13 (1−29)	20 (4−50)	75 (8−75)	50 (1−75)	< 0.001
Minor injury (NISS ≤ 8), n (%)	158 (88.3)	51 (30.0)	5 (8.5)	≤ 3 (≤ 2.6)	≤ 3 (12.5)	
Moderate injury (NISS 9 − 15), n (%)	14 (7.8)	59 (34.7)	13 (22.0)	≤ 3 (≤ 2.6)	≤ 3 (12.5)	
Severe injury (NISS 16 − 24), n (%)	7 (3.9)	48 (28.2)	19 (32.2)	7 (6.1)	≤ 3 (12.5)	
Critical injury (NISS 25 − 75), n (%)	0 (0)	12 (7.1)	22 (37.3)	106 (92.2)	19 (79.2)	< 0.001

NISS median values and categories were presented based on the column categories of survivors and fatalities. Differences between the distribution of NISS was estimated using Kruskal–Wallis test and differences between the categories were estimated using chi-squared test. Cell counts ≤3 are not reported in detail for confidentiality

The distribution of NISS categories also showed a strong association with the complexity of medical intervention required among survivors (Table 3). Minor injuries (NISS ≤ 8) were predominant among cases receiving no or only minimal interventions (88.3%), whereas this proportion declined markedly with increasing intervention level. Critical injuries (NISS 25–75) were absent among survivors with no/minimal interventions, but accounted for 7.1% of survivors with moderate interventions, and 37.3% of those requiring life-saving interventions.

Among fatalities, the majority were classified as critically injured (NISS 25–75) (92.2% of those found within 24 h and 79.2% of those found after 24 h) (Table 3). Only small numbers of fatalities fell into the minor injury (NISS ≤ 8), moderate injury (NISS 9 − 15), or severe injury (NISS 16 − 24) categories, and these were distributed across both early and late detection groups.

### Age, sex, and NISS predicting fatal injuries

For victims aged 60 years and older, there was no significant association with fatal injuries when adjusting for the NISS categories (Table S2); however, an association was indicated (OR 2.0, 95% CI 0.7–5.7). Adjusted for age categories and sex, the categories of moderate (NISS 9 − 15), severe (NISS 16 − 24), and critical injuries (NISS 25 − 75) were all conclusively associated with fatal injuries, using minor injuries (NISS ≤ 8) as a reference.

### Performance of NISS in predicting fatal injuries

Receiver operating characteristic (ROC) analysis demonstrated excellent discriminatory ability, with an AUC of 0.94 (95% CI 0.92–0.96, *p* < 0.001) for NISS in predicting fatal injuries (*n* = 174) using non-fatal injuries as reference (*n* = 349). Performance metrics at various NISS cut-offs are presented in Table S3. A good balance between sensitivity and specificity was observed around NISS thresholds of 12–29, where Youden’s Index exceeded 60%, with the highest value recorded at NISS ≥ 20 (72.1%, 95% CI 61.9–80.6). The specificity reached 100% at NISS cut-off ≥ 34, with a sensitivity of 59.2% (95% CI 51.5–66.6).

## Discussion

Our study addresses the lack of a standardised and quantifiable approach for forensic life-threat assessments by first categorising injury survivors into the complexity of medical interventions to be compared with autopsied cases; and secondly by analysing different NISS cut-off values for predicting fatal single stab injuries to the trunk.

Survived injuries requiring no or minimal medical interventions were mostly minor injuries (NISS ≤ 8) and would be unlikely to carry a considerable risk of death if the injuries were not treated. From a forensic perspective, such cases would generally be assessed as not life-threatening. In contrast, survived injuries that required life-saving interventions and injuries leading to death within 24 h were dominated by severe (NISS 16 − 24) and critical (NISS 25 − 75) injury categories, which would probably be fatal without timely medical intervention and thus fall within the forensic definition of ‘life-threatening injuries’.

Survived injuries that were not in an immediately life-threatening state when treated, classified as cases receiving moderate interventions, formed a heterogeneous group spanning all NISS categories. This reflects the overlap between injuries that may or may not be assessed as life-threatening in a forensic context. In particular, stab wounds penetrating the thoracic wall with minor pneumothoraces or haemothoraces were often managed promptly with drainage rather than observation, which obscures the natural progression of such injuries and complicates assessments of their natural course. A small subset of fatalities discovered after more than 24 h after the injury involved both critical injuries (such as ruptures of heart chambers or the aorta) and minor injuries with fatal complications at a later stage. The latter illustrates the complexity of assessing life-threat in injuries that are initially minor but if allowed to follow their natural course have the potential to progress to fatal outcomes; a scenario for which no international consensus guidelines address specifically (to our knowledge). Future research could focus on observing the rate of complications in the natural progression of initially minor injuries, to establish a more evidence-based risk assessment in these such cases.

In our material, injuries with documented internal organ or vessel injuries and injuries with perforations to thoracic cavity generally required at least moderate intervention among survivors. These findings align with established consensus guidelines, which would consider such injuries life-threatening [1, 5, 6]. A considerable proportion of injuries perforating the abdominal wall without damage to major organs or named vessels still required life-saving interventions or resulted in fatal outcomes, indicating a significant risk of death in the absence of treatment. Life-threatening haemorrhage from epigastric vessels has been previously described [17], and therefore trauma guidelines recommend observation for stable patients to detect delayed deterioration [18]. The high survival rate in hospitalised patients with stabs to abdomen [19, 20] may underestimate the natural course of abdominal wall injuries if left untreated, potentially shaping the empirical view that these injuries are not considered life-threatening in a forensic context.

The study results demonstrated excellent diagnostic performance of NISS in predicting fatal injuries. The risk of death within 24 h or the need for life-saving intervention increased consistently with higher NISS values, and a reasonable balance between sensitivity and specificity was observed around NISS thresholds of 12–29. These findings suggest that NISS, although developed to predict mortality in hospitalised patients, can be adapted to the forensic context to provide a quantitative and reproducible framework of assessing life-threat in single stab injuries to the trunk. However, it should be emphasised that in this study, NISS predicts fatal injuries, rather than directly measuring the forensic concept of a life-threatening injury. In forensic medicine, a life-threatening injury refers to injuries that would have carried a significant risk of death without any medical intervention, whereas a fatal injury in the present study refers to observed fatal outcome or expected directly fatal outcome in the absence of timely life-saving interventions. Accordingly, NISS thresholds and survivor categorisation by level of intervention should be interpreted as structured indicators that may support the forensic assessment of life-threat, rather than as a definitive criterion.

In forensic practice, the method could be applied through a stepwise workflow: (i) reviewing the documentation of injuries, the clinical condition of the injured patient, the medical interventions required, and potential complications, in order to perform; (ii) classification of injuries according to the AIS and calculation of the NISS score from the three most severe injuries; and (iii) interpretation of the NISS value together with consideration of the presumed natural course of the injuries in the absence of medical treatment, and with consideration of age and comorbidities when relevant.

In cases where the NISS indicates extensive anatomical injury, the causal relationship between trauma and death may often be relatively straightforward. In contrast, when the NISS is low or moderate, individual factors such as age and pre-existing comorbidities may play a more important role in determining the outcome. In such situations, the anatomical injury score alone may have a more limited explanatory value, and the forensic assessment necessarily relies more heavily on clinical context and expert interpretation. Thus, the evidential weight of the anatomical findings may vary depending on the overall injury severity reflected by the NISS.

In this way, the resulting NISS score could serve as a structured parameter supporting the forensic expert’s reasoning, with the potential to improve transparency of the assessments and consistency between experts. A schematic illustration of this workflow is presented in Figure S1.

There was a non-conclusive indication of an association between age (> 60 years) and fatal outcome, irrespective of NISS. This could indicate that age is an independent factor that needs to be considered when assessing whether an injury was life-threatening. This comes as no surprise, but the extent of the increased risk associated with age and at what point age becomes a factor meriting consideration requires further research. Also, age could potentially be a reflection on pre-existing health conditions, another factor that should be considered in the forensic assessment of injuries.

There are some previous studies that have applied trauma scores to evaluate forensic assessment of life-threatening injuries [6, 21, 22]. These studies established that ISS [6, 22], NISS [22], and the probability of survival trauma score [21] (a score that combines several anatomical and physiological factors [23]), can distinguish between life-threatening and not–life-threatening injuries in heterogeneous trauma populations. One of these studies reported that a NISS threshold of 14 achieved 82.8% sensitivity and 88.7% specificity when assessed as life-threatening by forensic pathologists [22]. Although our study population and outcome measures differed, our results were comparable, as our results demonstrated a NISS threshold of 14, yielding 88.5% sensitivity and 80.2% specificity in predicting fatal injuries. Unlike previous studies, our study integrated autopsy data with survivors requiring life-saving interventions to approximate the natural course of injuries instead of relying on empirically based forensic assessments as the outcome measure. This approach provides a more robust foundation for forensic life-threat assessments.

A key strength of this study lies in its nationwide scope, including both fatalities and survivors, with detailed information on internal injuries and medical interventions available through forensic registries. Also, we focused on a single, well-defined trauma mechanism—single stab injuries to the trunk—reducing confounding factors associated with multiple or diverse trauma types. However, the present model should therefore not be directly extrapolated to cases involving multiple stab wounds, other forms of penetrating trauma, or blunt trauma. Future studies should aim to validate the results in larger populations with different kinds of trauma.

Several limitations should also be acknowledged. The retrospective design relied on registry data, and in some cases, medical records did not cover the entire hospitalisation period, potentially reducing the precision of trauma scoring or leading to misclassification of intervention complexity. Additional factors, such as comorbidities and time before initiation of medical care, may also have influenced outcomes but could not be fully accounted for. Also, as NISS is based on anatomical injuries, it does not incorporate physiological parameters that may also play an important role in determining injury severity and outcome. Nevertheless, some relatively limited anatomical injuries may result in life-threatening or fatal outcomes, for example due to air embolism or delayed exsanguination from smaller vessels. Consequently, NISS should not be interpreted as a substitute for forensic expert judgement, but rather as a structured analytical aid that may support forensic assessment. Anatomical injury scoring systems alone may not always fully reflect the true severity of an injury.

Another methodological consideration relates to injury coding using the AIS, which forms the basis of the NISS calculation. In the Swedish Trauma Registry (SweTrau), formal AIS training is generally recommended to ensure consistent injury coding [24]. The authors of the present study did not undergo formal AIS training but both NISS raters are physicians with experience in injury assessment within their fields of forensic pathology and emergency medicine, respectively. To support consistent coding of complex injuries, consultation with an AIS instructor affiliated with the SweTrau was undertaken, particularly regarding the classification of cardiac injuries. While AIS training may represent a practical barrier for routine implementation of NISS in forensic practice, training programmes and collaboration with trauma registries may facilitate its broader application in forensic medicine. In a validation study of the SweTrau, the validity was found to be good, with 100% case completeness for NISS > 15 (major trauma causing severe or critical injuries). While the accuracy (exact agreement) of NISS was poor (38.3%), only three patients (2.5%) had a change in NISS that resulted in a change in category from not severe/critical to severe/critical injury or vice versa, which indicates that a cut-off of NISS > 15 for severely injured patients in SweTrau is valid [25].

## Conclusion

NISS demonstrated excellent performance in predicting fatal outcomes along the natural course of single stab injuries to the trunk, thereby addressing the lack of a quantitative and evidence-based framework in forensic life-threat assessments. Using NISS could provide consistency in forensic evaluations of life-threatening injuries, ultimately supporting fairness in the penal system. Nevertheless, as NISS, as it was used in this study, predicts mortality rather than a significant risk of death without medical intervention, its thresholds should be regarded as guidance rather than as definitive criteria.

## Supplementary Information

Below is the link to the electronic supplementary material.

Supplementary Material 1 (DOCX 16.1 KB)

Supplementary Material 2 (DOCX 16.1 KB)

Supplementary Material 3 (DOCX 18.9 KB)

Supplementary figure 1. Workflow for incorporating NISS into clinical practice of forensic assessment of life-threatening injuries. The evaluation begins with a review of injury documentation and the clinical course, followed by AIS coding and NISS calculation. The NISS should be interpreted together with the presumed natural course of the injury and individual factors such as age and comorbidities, forming the basis for the final forensic expert assessment. (PNG 117 KB)

High Resolution Image (TIF 103 KB)
