# Supplementary material for: Forensic life-threat assessments using trauma scoring in single stabs to the trunk
Source: Int J Legal Med. 2026 Apr 10;140(4):2555–64. doi: 10.1007/s00414-026-03781-6 (PMC13275780; doi:10.1007/s00414-026-03781-6)
Supplement: Supplementary file 1 — Supplementary Material 1 (DOCX 16.1 KB) [file 414_2026_3781_MOESM1_ESM.docx]

**Table S1. Example cases illustrating AIS coding and NISS calculation in single stab injuries to the trunk.**

| **Injury description** | **NISS calculation** | **NISS value** | **Interpretation** |
| --- | --- | --- | --- |
| Stab to the thorax only involving skin and subcutaneous fat (AIS 1), treated with sutures. | 1^2^ | 1 | Injury requiring minor intervention |
| Stab to the thorax penetrating the pleura causing a minor pneumothorax (AIS 2) and diaphragm laceration (AIS 2), requiring thoracic drainage and laparotomy with suturing of the diaphragm. | 2^2^ + 2^2^ | 8 | Injury requiring moderate intervention |
| Stab penetrating the thoracic wall damaging an intercostal artery (AIS 2), causing a minor lung laceration (AIS 3) with a major hemothorax (AIS 4), requiring thoracic drainage and blood transfusion. | 4^2^ + 3^2^ + 2^2^ | 29 | Injury requiring life-saving intervention |
| Stab to the abdomen with perforation of peritoneum and a minor perforation of the duodenum (AIS 2) with leakage of intestinal content causing peritonitis. | 2^2^ | 4 | Autopsy case, minor injury with fatal complication |
| Stab to the thorax, penetrating the sternum (AIS 2), pericardium (AIS 2) and the right cardiac chamber (AIS 6) causing hemopericardium. | 6* | 75 | Autopsy case, immediately fatal injury |

The NISS is calculated as the sum of the squares of the three most severe AIS injuries, regardless of anatomical region. AIS severity scores range from 1 (minor injury) to 6 (unsurvivable injury).

*According to standard NISS methodology, the presence of an AIS score of 6 automatically results in a NISS value of 75.

**Article title:** Forensic life-threat assessments using trauma scoring in single stabs to the trunk

**Journal name:** International Journal of Legal Medicine

**Author names:** Maria Berg von Linde, MD, Stefan Acosta, MD, PhD, Ardavan M. Khoshnood MD, PhD, Carl Johan Wingren, MD, PhD.

**Affiliation and e-mail address of the corresponding author:** Maria Berg von Linde, MD, Unit for Forensic Medicine, Department of Clinical Sciences Malmö, Faculty of Medicine, Lund University, Sweden. Electronic address: [maria.berg_von_linde@med.lu.se](mailto:maria.berg_von_linde@med.lu.se)
