# Supplementary material for: Forensic life-threat assessments using trauma scoring in single stabs to the trunk
Source: Int J Legal Med. 2026 Apr 10;140(4):2555–64. doi: 10.1007/s00414-026-03781-6 (PMC13275780; doi:10.1007/s00414-026-03781-6)
Supplement: Supplementary file 2 — Supplementary Material 2 (DOCX 16.1 KB) [file 414_2026_3781_MOESM2_ESM.docx]

**Table S2. Adjusted model with age, sex, and NISS categories as predictors of fatal injuries**

| Predictor | OR (95%CI) |
| --- | --- |
| <60 years, adjusted for sex and NISS categories | Ref |
| ≥60 years, adjusted for sex and NISS categories | 2.0 (0.7−5.7) |
|  |  |
| Males, adjusted for age and NISS categories | Ref |
| Females, adjusted for age and NISS categories | 0.6 (0.2−1.8) |
|  |  |
| Minor injury (NISS ≤8), adjusted for age and sex | Ref |
| Moderate injury (NISS 9−15), adjusted for age and sex | 6.5 (2.4−17.7) |
| Severe injury (NISS 16−24), adjusted for age and sex | 16.6 (6.5−42.5) |
| Critical injury (NISS 25−75), adjusted for age and sex | 369.5 (134.2−1017.4) |

Adjusted logistic regression model presenting associations between the predictors of age, sex, and NISS categories and the outcome of fatal injuries using non-fatal injuries as a reference.

**Article title:** Forensic life-threat assessments using trauma scoring in single stabs to the trunk

**Journal name:** International Journal of Legal Medicine

**Author names:** Maria Berg von Linde, MD, Stefan Acosta, MD, PhD, Ardavan M. Khoshnood MD, PhD, Carl Johan Wingren, MD, PhD.

**Affiliation and e-mail address of the corresponding author:** Maria Berg von Linde, MD, Unit for Forensic Medicine, Department of Clinical Sciences Malmö, Faculty of Medicine, Lund University, Sweden. Electronic address: [maria.berg_von_linde@med.lu.se](mailto:maria.berg_von_linde@med.lu.se)
