# Supplementary material for: Forensic life-threat assessments using trauma scoring in single stabs to the trunk
Source: Int J Legal Med. 2026 Apr 10;140(4):2555–64. doi: 10.1007/s00414-026-03781-6 (PMC13275780; doi:10.1007/s00414-026-03781-6)
Supplement: Supplementary file 3 — Supplementary Material 3 (DOCX 18.9 KB) [file 414_2026_3781_MOESM3_ESM.docx]

**Table S3.** **Diagnostic performance of NISS in predicting fatal injuries**

| NISS | Sensitivity (%) (95% CI) | Specificity (%) (95% CI) | Youden´s Index (%) (95% CI) |
| --- | --- | --- | --- |
| ≥1 | 100.0 (97.9–100.0) | 0.0 (0.0–1.1) | 0.0 (–2.1–1.1) |
| ≥4 | 100.0 (97.9–100.0) | 35.2 (30.2–40.5) | 35.2 (28.1–40.5) |
| ≥5 | 98.3 (95.0–99.6) | 53.6 (48.2–58.9) | 51.9 (43.2–58.5) |
| ≥8 | 98.3 (95.0–99.6) | 54.2 (48.8–59.5) | 52.5 (43.8–59.1) |
| ≥9 | 96.6 (92.7–98.7) | 59.9 (54.5–65.1) | 56.5 (47.2–63.8) |
| ≥10 | 94.3 (89.7–97.2) | 63.9 (58.6–68.9) | 58.2 (48.3–66.1) |
| ≥12 | 94.3 (89.7–97.2) | 71.6 (66.6–76.3) | 65.9 (56.3–73.5) |
| ≥13 | 93.7 (89.0–96.8) | 72.2 (67.2–76.8) | 65.9 (56.2–73.6) |
| ≥14 | 88.5 (82.8–92.8) | 80.2 (75.7–84.3) | 68.7 (58.5–77.1) |
| ≥16 | 88.5 (82.8–92.8) | 80.8 (76.3–84.8) | 69.3 (59.1–77.6) |
| ≥17 | 85.6 (79.5–90.5) | 82.0 (77.5–85.8) | 67.6 (57.0–75.8) |
| ≥18 | 82.2 (75.7–87.6) | 84.2 (80.0–87.9) | 66.4 (55.7–74.9) |
| ≥19 | 81.0 (74.4–86.6) | 89.7 (86.0–92.7) | 70.7 (60.4–79.3) |
| ≥20 | 81.0 (74.4–86.6) | 91.1 (87.6–93.9) | 72.1 (61.9–80.6) |
| ≥22 | 78.7 (71.9–84.6) | 91.1 (87.6–93.9) | 69.8 (59.4–78.5) |
| ≥24 | 74.7 (67.6–81.0) | 96.6 (94.1–98.2) | 71.3 (60.7–80.1) |
| ≥25 | 73.6 (66.4–80.0) | 96.6 (94.1–98.2) | 70.2 (59.6–79.0) |
| ≥26 | 71.8 (64.5–78.4) | 97.4 (95.2–98.8) | 69.2 (58.4–78.0) |
| ≥27 | 71.3 (63.9–77.9) | 98.0 (95.9–99.2) | 69.3 (58.4–78.5) |
| ≥29 | 66.1 (58.5–73.1) | 98.6 (96.7–99.5) | 64.7 (53.6–74.0) |
| ≥34 | 59.2 (51.5–66.6) | 100.0 (99.0–100.0) | 59.2 (48.0–66.6) |
| ≥36 | 50.6 (42.9–58.2) | 100.0 (99.0–100.0) | 50.6 (39.5–58.2) |
| ≥38 | 48.3 (40.7–56.0) | 100.0 (99.0–100.0) | 48.3 (37.2–56.0) |
| ≥41 | 45.4 (37.9–53.1) | 100.0 (99.0–100.0) | 45.4 (34.3–53.1) |
| ≥43 | 43.1 (35.6–50.8) | 100.0 (99.0–100.0) | 43.1 (32.0–50.8) |
| ≥45 | 41.4 (34.0–49.1) | 100.0 (99.0–100.0) | 41.4 (30.3–49.1) |
| ≥50 | 40.2 (32.9–47.9) | 100.0 (99.0–100.0) | 40.2 (29.1–47.9) |
| ≥57 | 38.5 (31.2–46.2) | 100.0 (99.0–100.0) | 38.5 (27.4–46.2) |
| ≥59 | 37.9 (30.7–45.6) | 100.0 (99.0–100.0) | 37.9 (26.8–45.6) |
| ≥66 | 37.4 (30.2–45.0) | 100.0 (99.0–100.0) | 37.4 (26.3–45.0) |
| 75 | 36.2 (29.1–43.8) | 100.0 (99.0–100.0) | 36.2 (25.1–43.8) |

Sensitivity, specificity, and Youden’s index were presented for different NISS cutoffs for predicting fatal injuries.

**Article title:** Forensic life-threat assessments using trauma scoring in single stabs to the trunk

**Journal name:** International Journal of Legal Medicine

**Author names:** Maria Berg von Linde, MD, Stefan Acosta, MD, PhD, Ardavan M. Khoshnood MD, PhD, Carl Johan Wingren, MD, PhD.

**Affiliation and e-mail address of the corresponding author:** Maria Berg von Linde, MD, Unit for Forensic Medicine, Department of Clinical Sciences Malmö, Faculty of Medicine, Lund University, Sweden. Electronic address: [maria.berg_von_linde@med.lu.se](mailto:maria.berg_von_linde@med.lu.se)
