# Supplementary figures and images for: Forensic life-threat assessments using trauma scoring in single stabs to the trunk
Source: Int J Legal Med. 2026 Apr 10;140(4):2555–64. doi: 10.1007/s00414-026-03781-6 (PMC13275780; doi:10.1007/s00414-026-03781-6)

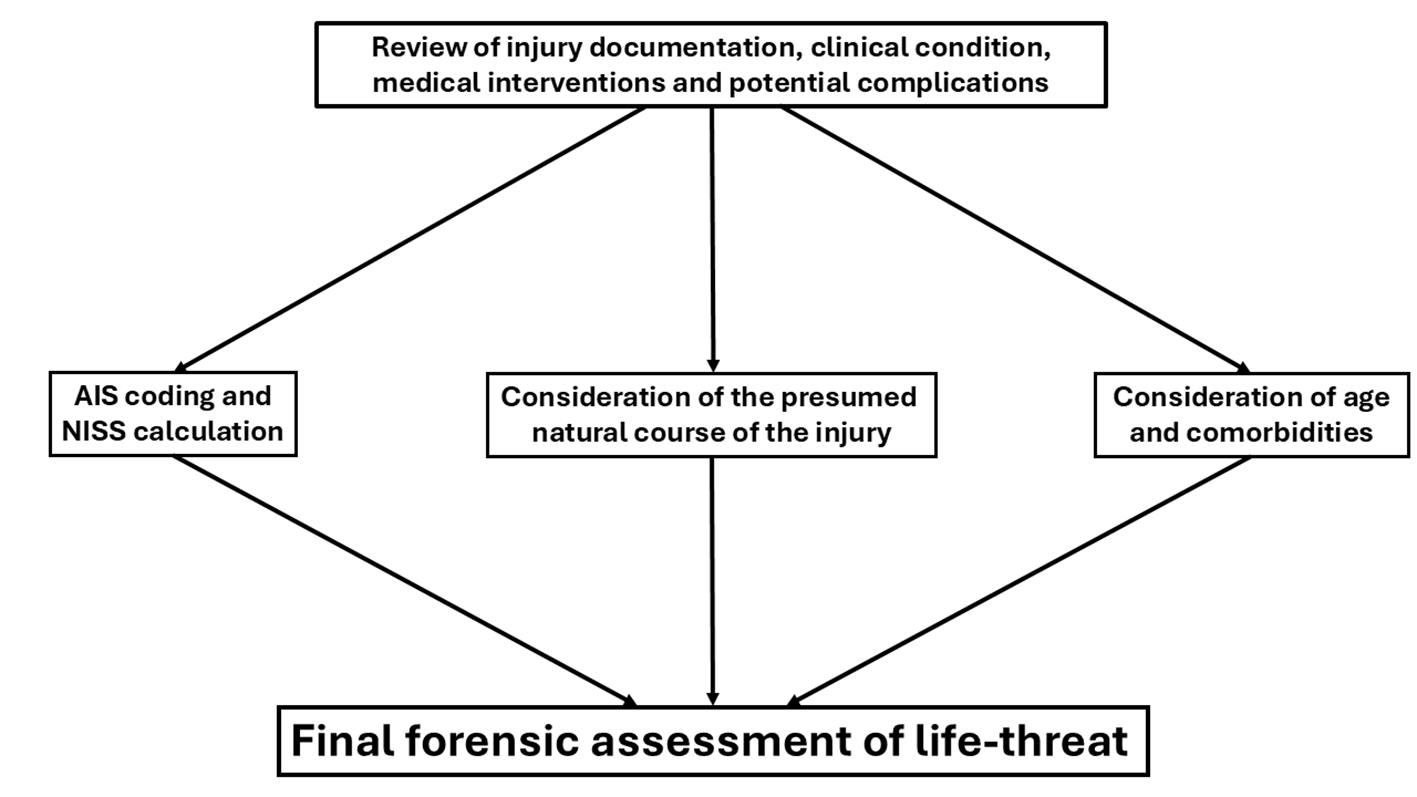

Supplement: Supplementary file 4 — Workflow for incorporating NISS into clinical practice of forensic assessment of life-threatening injuries. The evaluation begins with a review of injury documentation and the clinical course, followed by AIS coding and NISS calculation. The NISS should be interpreted together with the presumed natural course of the injury and individual factors such as age and comorbidities, forming the basis for the final forensic expert assessment. (PNG 117 KB) [file 414_2026_3781_Fig1_ESM.png]

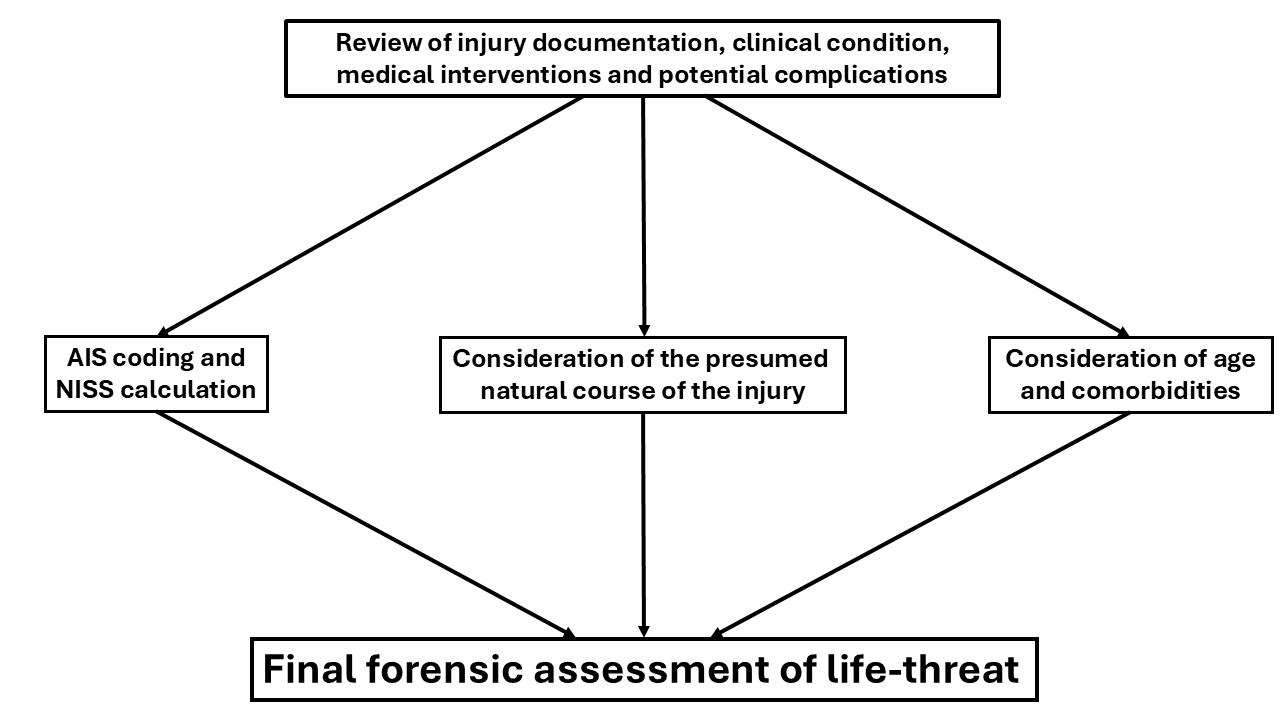

Supplement: Supplementary file 5 — High Resolution Image (TIF 103 KB) [file 414_2026_3781_MOESM4_ESM.tif]
